# Investigation of *CLOCK* rs1801260 Polymorphism of Circadian Genes in Schizophrenia Patients

**DOI:** 10.5152/eurasianjmed.2026.251049

**Published:** 2026-06-09

**Authors:** Patima Yekayeva, Burcu Türkgenç, Tayfun Gözler, İpek Yüksel, Korkut Ulucan, Nevzat Tarhan

**Affiliations:** 1 Department of Molecular Biology, Üsküdar University Graduate School of Science, İstanbul, Türkiye; 2 Department of Medical Biology, Department of Basic Medical Sciences, Üsküdar University Faculty of Medicine, İstanbul, Türkiye; 3 Department of Medical Genetics and Molecular Diagnostic Laboratory, Üsküdar University Faculty of Medicine, İstanbul, Türkiye; 4 Department of Basic Medical Sciences, Marmara University Faculty of Dentistry, İstanbul, Türkiye; 5 Department of Psychiatry, Department of Clinical Medical Sciences, Üsküdar University Faculty of Medicine, İstanbul, Türkiye; 6 Department of Psychiatry, NPISTANBUL Brain Hospital, İstanbul, Türkiye

**Keywords:** Allele frequency, circadian rhythm, genotype, polymorphism, schizophrenia

## Abstract

**Background:**

Schizophrenia is a mental condition characterized by abnormalities in emotional reactivity, thought processes, and perception. Recent studies have suggested a possible connection between the pathophysiology of schizophrenia and the *CLOCK* gene, a key element of the biological circadian rhythm. The *CLOCK* rs1801260 polymorphism has not yet been studied in a Turkish population with schizophrenia, and the existing data are contradictory. This study aimed to compare and interpret the *CLOCK* rs1801260 polymorphism in terms of demographic characteristics, genotype, and allele distribution in patients with schizophrenia and healthy controls.

**Methods:**

Deoxyribonucleic acid was purified from peripheral blood samples of 50 schizophrenia patients and 50 healthy control subjects, matched for age and sex. Genotyping was conducted by real-time polymerase chain reaction using the TaqMan Kit.

**Results:**

The odds of having the risk-associated TC or CC genotypes vs. the TT genotype were significantly higher in patients with schizophrenia compared to healthy controls (odds ratio (OR) = 2.92), and the risk C allele was also twice as prevalent in schizophrenia patients (OR = 2.00). The distributions of *CLOCK* rs1801260 genotypes (TT, TC, and CC) in the schizophrenia and the healthy control groups were independent of age and sex.

**Conclusion:**

The presence of the TC or CC genotypes, or having the homozygous or heterozygous C allele, may pose a risk for schizophrenia in the Turkish population. If confirmed in larger and more diverse samples, the *CLOCK *rs1801260 polymorphism may serve as a genetic marker for identifying at-risk individuals and guiding individualized treatment strategies aimed at stabilizing circadian rhythm.

**Main Points**

- The relationship between schizophrenia and the *CLOCK* rs1801260 gene polymorphism in a Turkish population was investigated for the first time in this study.
- Compared to the TT genotype, the odds of having the risk genotypes (TC and CC) were noticeably greater in patients with schizophrenia.
- In patients, the odds of carrying the risk-associated C allele were approximately twice those of the T allele, suggesting a possible connection between the C allele and schizophrenia.

## Introduction

Circadian rhythms are 24-hour changes in physiology and behavior that have evolved throughout phylogeny to assist species in predicting daily shifts in the environment, such as the light/dark cycle. The suprachiasmatic nucleus (SCN), located in the anterior portion of the hypothalamus, has a molecular clock that modifies circadian rhythms and is driven by the light-dark cycle. The molecular clock mechanism is composed of fundamental clock genes, such as circadian locomotor output cycles kaput* (CLOCK)*, *PER1*/*2*, *BMAL1*, *CRY1*, and *CRY2*,^1^^,^^2^ and it controls how the body’s internal and peripheral clocks synchronize.^3^ The *CLOCK* protein is a key regulator of circadian rhythm and controls many physiological processes.^4^^,^^5^ The *CLOCK* protein is expressed in the piriform cortex, hippocampus, hypothalamus, and SCN, and the gene is found on chromosome 4q12.^6^^,^^7^

The importance of circadian rhythm disruptions in the development, progression, and maintenance of mental diseases is increasingly recognized. Schizophrenia, a severe mental illness marked by psychotic symptoms and cognitive impairments,^8^ is strongly related to irregular circadian rhythms,^9^ which are thought to be a core chronobiological feature of the illness. Patients commonly exhibit sleep–wake disturbances, including altered melatonin rhythmicity and reduced sleep efficiency.^10^ Circadian rhythm disturbance is a hallmark of psychosis and is directly correlated with the severity of clinical symptoms. In addition, this impairment affects the patient’s quality of life.^11^ Although genetic predispositions are important, environmental factors also have an important influence on the origin and evolution of the disorder.^12^^,^^13^ Understanding these relationships is critical for developing successful preventive and therapeutic strategies.

According to previous studies, the molecular circadian *CLOCK* gene may be linked to the development of schizophrenia, and specific *CLOCK* gene polymorphisms may contribute to an individual’s susceptibility to the disorder, emphasizing the broader relevance of circadian rhythms in mental health. Specifically, the C allele of rs1801260 has been linked to a higher incidence of schizophrenia in the Japanese^14^ and Han Chinese populations.^15^ However, Kishi et al did not duplicate this finding in a larger Japanese group, assessing 6 *CLOCK* SNPs, including rs1801260.^16^ Similarly, studies conducted in Iranian^17^ and Caucasian^18^ populations have reported negative findings. These conflicting findings strongly suggest that the discrepancies stem from ethnicity and population-based genetic differences, emphasizing the critical need for further studies on different ethnic groups.

To date, the *CLOCK *rs1801260 polymorphism has not been investigated in a Turkish population with schizophrenia. To address this critical gap and provide valuable information, considering the genetic diversity among populations and the mounting data that correlate circadian rhythm disruptions with psychiatric conditions, in the current study, the study aimed to compare and interpret the *CLOCK *rs1801260 polymorphism, a circadian gene, in terms of demographic characteristics, genotype, and allele distributions in patients with schizophrenia and healthy controls. This study furthers the understanding of the genetic basis of schizophrenia and the possible involvement of circadian genes in disease susceptibility. Ultimately, clarifying this particular genetic relationship may provide a preliminary framework for the creation of risk assessment tools or individualized treatment strategies for schizophrenia patients based on their chronobiological profile.

## Material and Methods

### Collection of Study Samples

This study was approved by the Üsküdar University non-interventional research ethics committee (Approval no: 61351342/2020-169, Date: March 27, 2020) under the principles of the Declaration of Helsinki. Written informed consent was obtained from all participants, and for those under the age of 18 years, consent was provided by their parents or legal guardians. The recruitment period for the study was January 2022-June 2024. Fifty patients aged 16-63 who had been admitted to the psychiatry outpatient clinic of NPISTANBUL Brain Hospital were followed up as inpatients or outpatients in the psychiatry service and diagnosed with schizophrenia based on DSM-5 diagnostic criteria,^19^ which are still used in DSM-5-TR,^20^ by a senior psychiatry resident and a psychiatry specialist through a comprehensive clinical interview. The schizophrenia patient group comprised individuals at different stages of illness at the time of inclusion, including acute psychotic episodes, partial or full remission, and clinically stable chronic phases. Severe mental illnesses that coexist with schizophrenia, such as major depressive disorder or bipolar disorder, were excluded from the schizophrenia group. Fifty healthy control subjects between the ages of 16 and 48 who had no personal or family history of psychotic disorders were included in the study. Exclusion criteria for schizophrenia and the healthy control groups included the circadian rhythm sleep–wake disorders, primary sleep disorders (e.g., insomnia, obstructive sleep apnea) that could affect the circadian rhythm regulation. To reduce potential confounding factors, the patient and healthy control groups were matched for age and sex. Blood samples from 100 volunteers were collected in 1 cc tubes containing 2% ethylenediaminetetraacetic acid.

### Deoxyribonucleic Acid Isolation and Genotyping Analysis

Genomic deoxyribonucleic acid (DNA) was isolated from blood samples using the PureLink Genomic DNA Kit (Invitrogen, USA), following the manufacturer’s instructions. Following isolation, the concentration and purity of the DNA samples were checked and stored at −20°C. After DNA isolation, the *CLOCK* rs1801260 polymorphism was analyzed in 50 patient samples and 50 healthy control samples by real-time polymerase chain reaction (PCR) using a TaqMan Kit specifically designed for the study. For the *CLOCK* T3111C (rs1801260) SNP genotyping assay, 2.5 μL of the Applied Biosystems TaqPathTM Master Mix (Thermo Fisher Scientific, USA), 0.50 μL of TaqMan SNP Genotyping Assays, human rs1801260 (Thermo Fisher Scientific, USA), 1.75 μL of distilled water, and 1 μL of DNA were added to the mixture to make a total of 5.75 μL of reaction mix. The Applied Biosystems QuantStudio 5 Real-Time PCR System (Thermo Fisher Scientific, USA) was used for real-time PCR. All PCRs were performed under the following thermal cycler conditions: a pre-read phase of 30 seconds at 60°C, a denaturation hold phase of 5 minutes at 95°C, 40 cycles of a PCR phase of 15 seconds at 95°C and 1.30 minutes at 60°C, followed by a post-read phase of 15 seconds at 60°C and a ramp rate of 1.6°C/s. Genotyping calls were conducted automatically using the desktop software.

### Statistical Analysis

All statistical analyses were evaluated using SPSS Statistical Software v.25 (IBM Corporation, Armonk, NY, USA). Demographic, clinical, and *CLOCK* rs1801260 polymorphism categorical data (n and %) and numerical data are presented as mean ± SD. The Kolmogorov–Smirnov test was used to assess the normality of the data distribution. Since the Kolmogorov–Smirnov value was determined as *P* > .05, the unpaired *t*-test, a parametric test, was used for comparisons between the patient and healthy control groups. The analysis of variance test was used to compare the mean age across the different genotype groups. If a significant result was found between groups, the post-hoc Bonferroni test was applied to identify which groups differed significantly. Fisher’s exact tests and Pearson’s chi-square test were utilized to compare categorical variables. To evaluate population genetic validity, the healthy control group was subjected to the Hardy–Weinberg Equilibrium (HWE) assessment using Pearson’s chi-square test. Statistical significance was set at *P* < .05.

## Results

The comparison of age at diagnosis, sex, and *CLOCK* rs1801260 genotype distribution between 50 schizophrenia patients and 50 healthy controls is shown in Table 1. The schizophrenia group had a mean age of 36.26 ± 11.26 years and a median age of 35.5 years, with an age range of 16-63 years. The healthy control group had a mean age of 34.04 ± 7.09 years and a median age of 32.5 years, with an age range of 16 to 48 years. There was no statistically significant difference between the groups when considering the age (*P* = .13) or sex distribution (*P* = .69), confirming that the cohorts were well-matched.

The distribution of the genotypes of the* CLOCK* rs1801260 polymorphism in the schizophrenia group was 24% (n = 12) TT, 62% (n = 31) TC, and 14% (n = 7) CC, whereas genotype frequencies in the healthy controls were 48% (n = 24) TT, 46% (n = 23) TC, and 6% (n = 3) CC. The overall difference in genotype distribution between the 2 groups was statistically significant (*P* = .034). This difference suggests a higher frequency of the TC and CC genotypes in the schizophrenia group compared with healthy controls. The *CLOCK *rs1801260 genotype distribution in the healthy control group was found to be in accordance with HWE (*P* = .20). This result indicates that the genetic structure of the healthy control group is balanced in terms of population genetics (Table 1).

As demonstrated in Table 2, under a dominant model, risk-associated genotypes (TC or CC) were significantly more prevalent in the schizophrenia patients than in healthy controls (76.0% vs. 52.0%, *P* = .012). Accordingly, the odds of having TC or CC risk genotypes in patients were 2.92 times higher than those of the TT genotype compared to healthy control subjects (OR = 2.92, 95% CI: 1.28-6.59). Allelic distribution analysis showed that the C risk allele was markedly more frequent in the schizophrenia group (45.0%) than in the healthy control group (29.0%), demonstrating a statistically significant difference in allele distribution between the groups (*P *= .019). As a primary result of the study, the odds of carrying the C allele were approximately 2 times higher in patients with schizophrenia (OR = 2.00, 95% CI: 1.14-3.65) (Table 2).

When the *CLOCK* rs1801260 genotypes (TT-Normal, TC-Risk, and CC-Risk) were compared separately with respect to age distribution within the 2 groups, no statistically significant differences in mean age were observed among genotypes in either the schizophrenia (*P* = .41) or the healthy control group (*P* = .060). Similarly, despite differences in the proportion of males and females across genotypes, sex distribution did not differ significantly across genotypes in the schizophrenia group (*P* = .66) or the healthy controls (*P* = .96) (Table 3). These findings show that the observed genetic associations are not confounded by the participants’ age or gender.

## Discussion

Alterations in core clock genes such as *CLOCK* have been associated with schizophrenia, indicating a genetic propensity for circadian rhythm disturbances. Individuals with schizophrenia frequently exhibit severe circadian misalignment, including non-24-hour sleep-wake cycles, which can exacerbate mental symptoms.^21^ Studies have shown circadian variability in auditory hallucinations^22^ and reduced clock gene expression in blood and skin cells,^23^^,^^24^ emphasizing the relevance of circadian regularity in mental health.

Despite contradictory results in other populations,^14^^-^^17^ the CLOCK rs1801260 polymorphism remains a key candidate for study, particularly as it has not yet been investigated in the Turkish population. The study sought to explore and interpret the *CLOCK* rs1801260 polymorphism, a circadian gene, regarding demographic features, genotype, and allele distribution in schizophrenia patients and healthy controls.

This study included 50 individuals with schizophrenia (mean age: 36.26 ± 11.26 years) and 50 healthy controls (mean age: 34.04 ± 7.09 years). There were no notable differences in age or sex between groups, confirming a well-matched cohort. Although schizophrenia onset and prevalence often show sex-specific variations due to environmental and hormonal factors,^25^^,^^26^ the homogeneity of the groups supports that the observed genetic connections are not confounded by these demographic variables.

The most significant finding of the study was the distribution of the *CLOCK* genotypes, which emerged as a potential genetic risk factor for schizophrenia. Specifically, carriers of TC and CC genotypes had nearly 3 times the odds of developing schizophrenia compared to those with the TT genotype (OR = 2.92, 95% CI: 1.28-6.59, *P* = .012). Furthermore, confirming this result, the odds of having the C allele in schizophrenia patients were 2-fold higher than those of the T allele (OR = 2.00, 95% CI: 1.14-3.65, *P* = .019). Crucially, these genetic associations remained independent of age and sex, with the adherence to HWE (*P *= .20), further suggesting that the findings are unlikely to be influenced by genotyping errors. This strengthens the evidence for a potential biological link between the *CLOCK* rs1801260 polymorphism and schizophrenia.

**Figure 1:**
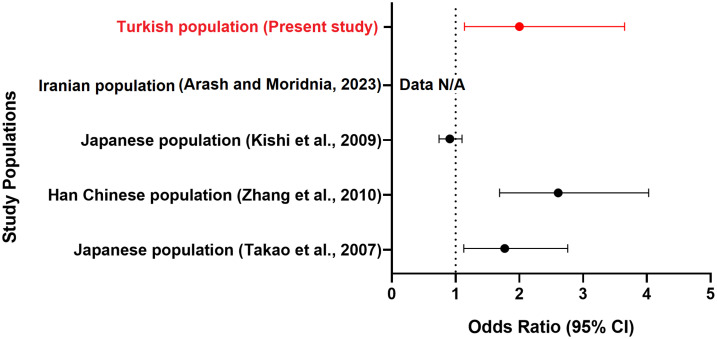
Comparison of the *CLOCK* rs1801260 polymorphism associations with schizophrenia across different populations. The odds ratios (OR) and 95% CI are shown for the present study (Turkish population, highlighted in red) and previously reported studies in East Asian populations. The vertical dashed line represents the null effect (OR = 1.0). Odds ratio > 1.0 indicates an increased risk associated with the C allele. Notably, Arash and Moridnia (2023) reported no significant association in an Iranian population, but the study lacked the necessary allele frequencies or OR values for inclusion in this forest plot. N/A, Not available.

The findings in the Turkish population demonstrate a similar risk profile to previous positive reports in East Asian cohorts, such as Japanese^14^ and Han Chinese^15^ populations, where the C allele was associated with increased susceptibility to schizophrenia (Figure 1). As illustrated in the forest plot, the OR and 95% CI of the study align with these associations. However, the global literature remains contradictory, as a large-scale Japanese study^16^ and a recent Iranian study^17^ failed to find a significant association for the rs1801260 locus. Notably, the Iranian study could not be included in Figure 1 because the necessary allele frequencies or OR values were unavailable. These discrepancies suggest that the impact of *CLOCK* gene variants may be modulated by “genetic heterogeneity” and population-specific genetic backgrounds. The findings contribute to the growing body of evidence suggesting an association within this global debate, emphasizing the importance of ethnicity-specific genetic research.

Genetic changes in the *CLOCK* gene mechanism may contribute to individuals developing psychiatric disorders similar to external disruption of the clock system. Since many psychiatric diseases are related to sleep disruptions, stabilizing the circadian system with regular sleep-wake cycles and light therapy has been reported to be an affordable therapeutic approach.^27^

However, this study has several limitations that need to be addressed in subsequent studies. The comparatively small sample size (50 healthy controls and 50 patients) restricted the generalizability of the results, and the analysis was limited to 1 genetic variant. In addition, only simple demographic variables, such as age and sex, could be compared between groups. Furthermore, clinical variables, such as disease subtype, symptom severity (e.g., PANSS/BPRS scores), illness duration, or treatment history (e.g., antipsychotic type/dose) were not included in the comparison. In light of the marked clinical heterogeneity observed in schizophrenia, future studies should aim to incorporate more precise phenotypic characterizations and subdivide patient groups according to their clinical course or symptom profiles, as well as the differential gene expression within clinical subgroups. Furthermore, the chronotype, an individual’s natural preference for morningness or eveningness, is a key behavioral expression of the underlying molecular circadian rhythm. In both healthy and clinical populations, the *CLOCK* rs1801260 polymorphism itself has been repeatedly linked to chronotype variation, where the risk C allele is often associated with a predisposition for eveningness.^28^^,^^29^ To connect the reported genetic risk (the rs1801260 polymorphism) with the clinically significant behavioral phenotype (circadian disruption) in schizophrenia patients, chronotype assessment would be essential in subsequent research to determine if risk-allele carriers respond differently to rhythm-stabilizing protocols. Observing how these polymorphisms affect clinical data or gene expression would provide a deeper correlation between the genetic findings and biological and clinical causes. Such analysis might help identify clinically meaningful subgroups and refine individualized therapeutic approaches in schizophrenia. It is believed that this initial finding in the Turkish population will inspire future molecular and clinical studies.

In conclusion, this study provides the first evidence in a Turkish cohort that carriers of the *CLOCK* rs1801260 TC or CC genotypes or the C allele have significantly increased odds of schizophrenia, supporting the involvement of circadian rhythm disruptions in the etiopathogenesis of schizophrenia. If confirmed in larger and clinically diverse samples, this polymorphism could serve as a genetic marker to identify patients who would benefit most from treatments aimed at restoring circadian rhythm stability. Future research should investigate whether this genetic variant can tailor personalized schizophrenia management through interventions such as targeted light therapy to restore biological clock homeostasis in at-risk individuals.

## Figures and Tables

**Table 1.** Comparison of Demographic and Genetic Characteristics Between Schizophrenia and Healthy Control Groups

**Variables**	**Schizophrenia**	**Healthy Control**	***P***
Age at diagnosis (years)			
Mean ± SD	36.26 ± 11.26	34.04 ± 7.09	.13*
Median (min-max)	35.5 (16-63)	32.5 (16-48)	—
Sex, n (%)			
Male	28 (56.0)	30 (60.0)	.69^†^
Female	22 (44.0)	20 (40.0)	
Genotype - phenotype for *CLOCK* rs1801260 (n, %)			
TT - Normal	12 (24.0)	24 (48.0)	**.034** ^†^
TC - Risk	31 (62.0)	23 (46.0)	
CC - Risk	7 (14.0)	3 (6.0)	

The genotype distribution of the healthy control group’s CLOCK rs1801260 was found to be in accordance with the Hardy–Weinberg equilibrium (HWE) (*P* > .05).

*Unpaired t-test

^†^Pearson chi-square test, *P* < .05 statistically significant.

**Table 2.** The Genotype-Phenotype and Allele Distributions of the *CLOCK* Gene

**Genotype-Phenotype**	**Schizophrenia** **n (%)**	**Healthy Control** **n (%)**	***P*** *	**Odds Ratio (OR)**	**(95% CI)**
rs1801260					
TT - Normal	12 (24.0)	24 (%48.0)	**.012**	2.92	(1.28-6.59)
TC or CC - Risk genotype	38 (76.0)	26 (52.0)			
Allele					
T	55 (55.0)	71 (71.0)	**.019**	2.00	(1.14-3.65)
C	45 (45.0)	29 (29.0)			

*Pearson chi-Square test, *P* < .05 statistically significant (shown in bold).

**Table 3.** Comparison of the *CLOCK* Genotypes with Age and Sex Between Schizophrenia and Healthy Control Groups

**Variables**	**TT**	**TC**	**CC**	***P***
Schizophrenia - *CLOCK* rs1801260				
Age at diagnosis (mean ± SD)	36.0 ± 7.51	38.3 ± 7.52	35.7 ± 9.15	.41*
Sex, n (%)				
Male	7 (58.3)	16 (51.6)	5 (71.4)	.66^†^
Female	5 (41.7)	15 (48.4)	2 (28.6)	
Healthy control - *CLOCK* rs1801260				
Age at diagnosis (mean ± SD)	31.25 ± 8.14	32.2 ± 6.12	35.0 ± 3.46	.060*
Sex, n (%)				
Male	14 (58.3)	14 (60.9)	2 (66.7)	.96^†^
Female	10 (41.7)	9 (39.1)	1 (33.3)	

*ANOVA test.

^†^Fisher’s exact test, *P* < .05 statistically significant.

## Data Availability

The data that support the findings of this study are available on request from the corresponding author.
